# Corrosive extracellular polysaccharides of the rock-inhabiting model fungus *Knufia petricola*

**DOI:** 10.1007/s00792-017-0984-5

**Published:** 2017-12-23

**Authors:** Romy Breitenbach, Dorothee Silbernagl, Jörg Toepel, Heinz Sturm, William J. Broughton, Guilherme L. Sassaki, Anna A. Gorbushina

**Affiliations:** 1 Department 4 (Materials and Environment), Bundesanstalt für Materialforschung und -prüfung (BAM), Unter den Eichen 87, 12205 Berlin, Germany; 2 Department of Biology, Chemistry and Pharmacy, Freie Universität Berlin, Takustr. 3, 14195 Berlin, Germany; 3 Division 6.6 (Nanotribology and Nano-Structuring), Bundesanstalt für Materialforschung und -prüfung (BAM), Unter den Eichen 87, 12205 Berlin, Germany; 4 Department of Solar Materials, Applied Biocatalytics, Helmholtz Centre for Environmental Research, Permoserstraße 15, 04318 Leipzig, Germany; 5 Institute of Machine Tools and Factory Management, TU Berlin, Pascalstr. 8-9, 10587 Berlin, Germany; 6 Department of Biochemistry and Molecular Biology, Federal University of Parana, Curitiba, PR CP 19046 Brazil; 7 Department of Earth Sciences, Freie Universität Berlin, Malteserstraße 74-100, 12249 Berlin, Germany

**Keywords:** EPS, Corrosion, α-1,4- and α-1,6-glucans, Pullulan, Melanised microcolonial fungi (MCF), Sub-aerial biofilms (SAB)

## Abstract

Melanised cell walls and extracellular polymeric matrices protect rock-inhabiting microcolonial fungi from hostile environmental conditions. How extracellular polymeric substances (EPS) perform this protective role was investigated by following development of the model microcolonial black fungus *Knufia petricola* A95 grown as a sub-aerial biofilm. Extracellular substances were extracted with NaOH/formaldehyde and the structures of two excreted polymers studied by methylation as well as NMR analyses. The main polysaccharide (~ 80%) was pullulan, also known as α-1,4-; α-1,6-glucan, with different degrees of polymerisation. Αlpha-(1,4)-linked-Glc*p* and α-(1,6)-linked-Glc*p* were present in the molar ratios of 2:1. A branched galactofuromannan with an α-(1,2)-linked Man*p* main chain and a β-(1,6)-linked Gal*f* side chain formed a minor fraction (~ 20%). To further understand the roles of EPS in the weathering of minerals and rocks, viscosity along with corrosive properties were studied using atomic force microscopy (AFM). The kinetic viscosity of extracellular *K. petricola* A95 polysaccharides (≈ 0.97 × 10^−6^ m^2^ s^−1^) ranged from the equivalent of 2% (w/v) to 5% glycerine, and could thus profoundly affect diffusion-dominated processes. The corrosive nature of rock-inhabiting fungal EPS was also demonstrated by its effects on the aluminium coating of the AFM cantilever and the silicon layer below.

## Introduction

Microbial biofilms, one of the most successful forms of life, are held together by an extracellular matrix (Flemming et al. 2016). Almost all microbial groups, including fungi, that produce extracellular polymeric substances (EPS) tend to form biofilms (Blankenship and Mitchell 2006). Fungal cell walls contain chitin and β-glucans surrounded by a complex network of polysaccharides, glycoproteins and enzymes (Barreto-Bergter and Figueiredo 2014). Extracellular polysaccharides produced by fungi (both filamentous- and yeast-like ones) range from glucans to galactans including *N*-acetylhexosamines, mannans and galactomannan complexes. Many predominant cell-wall polysaccharides in fungi are soluble and can actively participate in EPS formation (reviewed in Breitenbach et al. 2017). Due to a wide variety of biotechnological and medical applications, the α-glucan pullulan and the β-glucan schizophyllan are probably the best characterised fungal extracellular polysaccharides (Prajapati et al. 2013; Zhang et al. 2013). This work is dedicated exclusively to the largely unknown EPS formed by rock-inhabiting fungi.

Rock-inhabiting microcolonial fungi (MCF) and black yeasts (orders Chaetothyriales and Dothideomycetes) are amongst the most stress-tolerant eukaryotes known (Gorbushina 2003; Zakharova et al. 2013). Black slow-growing fungi thrive in stressful habitats including hot and cold deserts, rock surfaces (Ruibal et al. 2008; Selbmann et al. 2005) and on material surfaces (Gorbushina 2007; Gorbushina et al. 1993; Ragon et al. 2011). Melanised fungi are also important opportunistic human pathogens (Chowdhary et al. 2015)—and are descendants of the rock-inhabiting taxa that occupy the basic clades of Chaetothyriales and Dothideomycetes (Gueidan et al. 2008; Ruibal et al. 2008). Amongst the most important stress-reduction strategies of MCF are thick melanised cell walls and EPS (Gorbushina et al. 2008; Kuncheva et al. 2013; Pavlova et al. 2011; Selbmann et al. 2005). Production of diverse EPS enables organisms to survive various stresses including temperature fluctuations, low water availability, UV-radiation, nutrient deficiency and even outer space (Onofri et al. 2012; Scalzi et al. 2012; Selbmann et al. 2014). EPS are also involved in interactions of MCF with algae, cyanobacteria and heterotrophic bacteria (Gorbushina and Broughton 2009). EPS of rock-inhabiting bacteria and MCF actively participate in; (a) weathering and soil formation by interaction with substrates; (b) enhance the interaction with symbiotic partners and; (c) increase stress tolerance by retaining water and inhibiting access of external agents to the biofilms (Gadd 2007; Gorbushina and Broughton 2009; Tourney and Ngwenya 2014). To improve our understanding of interactions that guarantee success of MCF in natural environments, we selected the melanised fungus *Knufia petricola* A95 as a model rock-inhabiting fungus (order *Chaetothyriales*) (Gorbushina and Broughton 2009; Nai et al. 2013; Noack-Schonmann et al. 2014). In earlier work, we had shown that *K. petricola* A95 produces a distinct EPS layer (Gorbushina et al. 2008), but methods to extract and characterise the EPS were needed. Here, we present methods for the quantitative isolation of EPS, for the structural determination of EPS of melanised MCF including those of *K. petricola* A95 and show images of *K. petricola* A95 EPS under the scanning electron microscope. Extracts of *K. petricola* A95 EPS were also used to determine material-relevant properties including viscosity and corrosivity.

## Materials and methods

### Fungal strains and growth conditions

*Knufia petricola* A95 was isolated from a marble monument in Athens (Greece) (Wollenzien et al. 1997). It is maintained at the Centraalbureau voor Schimmelcultures (as CBS 123,872) in Utrecht, The Netherlands and the Federal Institute for Materials Research and Testing (A95) in Berlin, Germany. *K. petricola* A95 was cultivated in liquid malt-extract broth (MEB). SABs were grown on cellulose acetate filters (0.45 µm; Sartorius, Göttingen, Germany) placed on malt-extract agar plates (MEA). After mechanical separation (Nai et al. 2013), 1 ml of the cell suspension was spread onto the filter and incubated at 25 °C for 7 days.

### Extraction and purification of exopolysaccharides

Biofilms were scraped-off three individual filters. Triplicates were pooled and suspended in PBS buffer (10 ml). EPS were extracted from biofilm suspensions according to Liu and Fang (2002) with modifications. After addition of formaldehyde (100 µl), the biofilm suspension was incubated for 1 h at 4 °C. Then, 4 ml of 1 M NaOH were added and incubated at ambient temperature for further 3 h. After centrifugation (7190×*g*, 10 min), the supernatant was transferred to a new tube. Ice-cold absolute ethanol was added to a final concentration of 70% (v/v), mixed thoroughly and incubated at 4 °C for 16 h to precipitate the polysaccharides. Light microscopy was used to ensure that the cells were not damaged during extraction. The precipitate was washed twice with 70% (v/v) ethanol, dried at 45 °C, weighed, re-suspended in deionised water and filter sterilised (cellulose acetate syringe filters; 0.2 µm; Sartorius). All extractions were performed in triplicate and the experiment was repeated three times. The phenol–sulphuric acid method with glucose as the standard was used to quantify total carbohydrates in the extracts (Dubois et al. 1956).

### Monosaccharide composition

Monosaccharides were determined as their alditol acetate derivatives according to Albersheim et al. (1967) with modifications. Extracellular polysaccharides (50 µg) and inositol (5 µg) as the internal standard were hydrolysed with 2 M TFA (200 µl) at 100 °C for 3 h, cooled to room temperature and evaporated to dryness. 50% (v/v) 2-propanol was added to remove TFA. Monosaccharides were reduced overnight with NaBD_4_ (1 mg) in 1 M ammonium hydroxide, neutralised with acetic acid and evaporated to dryness (methanol was added to decompose excess NaBD_4_). The reduced monosaccharides were acetylated with pyridine and acetic anhydride [100 µl; 1:1 (v/v)] at 100 °C for 1 h. Then, the alditol acetates were evaporated to dryness, re-suspended in acetone and identified by retention times as well as electron impact profiles. A mixture of d-galactose, d-glucose and d-mannose with inositol was used as the standard.

### Methylation analyses

Permethylation of polysaccharides was performed using a modified method of Ciucanu and Caprita (2007). Total polysaccharide (2 mg) was dissolved in deionised water (20 µl), DMSO (900 µl) was added and the sample was sonicated for 45 min. Powdered NaOH (30 mg) and an excess of solid NaOH (60 mg) were added to each sample which were again sonicated for 45 min, then stirred at room temperature for another 45 min. Methyl iodide (350 µl) was added and the solutions were sonicated for 30 min. More methyl iodide (100 µl) was then added and the mixtures stirred at room temperature for a further 45 min. Permethylated polysaccharides were extracted with chloroform, the organic phase was washed three times with deionised water and evaporated to dryness. Thereafter, the samples were hydrolysed, reduced and acetylated as described above (preparation of alditol acetates). Identification of partially methylated alditol acetates was based on retention times as well as mass fragmentation patterns of standards synthesised as described subsequently.

### Preparation of PMAA standards

Permethylated alditol acetate (PMAA) standards were synthesised according to Sassaki et al. (2005) with the modifications of Wang et al. (2007). Briefly, d-galactose, d-glucose and d-mannose (each 60 mg) were refluxed in 2% MeOH–HCl at 70 °C for 16 h, neutralised with an excess of NaHCO_3_, filtered and evaporated to dryness. Methyl glycosides were dissolved in DMF (2 ml) with BaO (200 mg), Ba(OH)_2_·8 H_2_O (10 mg) and methyl iodide (1 ml). Samples were stirred in the dark and aliquots (300 µl) were removed at 1 h intervals, evaporated to dryness and derivatised to PMAAs as described above (preparation of alditol acetates). After 3 h for glucose and mannose (4 h for galactose) aliquots contained all substitution patterns necessary for analysis and were used for PMAA identification of exopolysaccharides. Linkage types of the synthesised PMAAs were assigned by comparison with mass spectra (Sassaki and Souza 2013).

### Gas chromatography–mass spectrometry (GC–MS) analyses

GC–MS analyses were performed using a 6890N Network Gas Chromatograph linked to a 5973 Network mass selective detector (Agilent, Santa Clara, CA, USA) with He as the carrier gas. Permethylated alditol acetates were injected onto a fused silica HP-5MS capillary column (30 m × 0.25 mm × 0.25 µm). Chromatographic conditions: injector temperature—220 °C; injection volume—1 µl; temperature of the ion-source—230 °C and temperature of the quadrupole—150 °C; acquiring mode—scan, from *m*/*z* 40 to *m*/*z* 500. Alditol acetates were analysed using the following temperature programme: 100 °C, held for 2 min, 100 to 180 °C at 10 °C min^−1^, held for 2 min, 180 °C to 190 °C at 1 °C min^−1^, held for 4 min, 190 °C to 240 °C at 10 °C min^−1^, held for 5 min, then to 280 °C at 20 °C min^−1^, held for 5 min. PMAA were analysed with the temperature programme: 80–120 °C at 5 °C min^−1^, held for 1 min; 120–230 °C at 3 °C min^−1^, held for 1 min; to 280 °C at 20 °C min^−1^, held for 5 min.

### Fourier transform infrared (FTIR) spectroscopy

For FTIR spectroscopy, 10 µl of EPS solution was placed on a ZnSe-crystal (Korth Kristalle GmbH, Kiel, Germany) and dried at 45 °C for 30 min. The infrared spectra were recorded with a FTIR spectrometer (Vertex 70, Bruker Optics, Karlsruhe, Germany) coupled with an infrared microscope (Hyperion 3000, Bruker Optics). The spectra were measured in the absorbance mode in the frequency range 4000–800 cm^−1^ with a spectral resolution of 4 cm^−1^. Spectra were baseline corrected and the CO_2_ peak removed. The mean value of at least five spectra was calculated.

### Nuclear magnetic resonance spectroscopy

Extracellular polysaccharides (2 mg) were dissolved in 200 µl of D_2_O and samples loaded into 5 mm Shigemi tube (Shigemi Inc, Allison Park, PA, USA). The nuclear magnetic resonance spectra were obtained using a Bruker Avance III 14.1 T spectrometer equipped with an inverse 5-mm probe head (QXI) at 323 K. 1D ^1^H-NMR at 600 MHz were performed after 90° (p1) pulse calibration. Pre-saturation of residual HDO was carried out with the pulse programme zgpr, using a relaxation delay = 1.0 s, number of time domain points = 65,536 and acquisition time = 5.1 s to obtain a spectral width of 6393 Hz, using 32 data points. ^1^H and ^13^C chemical shifts were determined by 2D-NMR analyses using HSQC, COSY and DOSY. HSQC hetero-nuclear correlations via double inept transfers with decoupling during acquisition, using sensitivity improvement trim pulses in inept transfer and shaped pulses for all 180° pulses on the ^13^C channel, complied in the pulse programme hsqcetgpsisp2.2 using 6393 Hz (^1^H) and 24,900 Hz (^13^C) widths and a recycle delay of 1.160 s. The spectra were recorded for quadrature detection in the indirect dimension, using 16 scans per series of 2048 × 256 data points with zero filling in F1 (2048) prior to Fourier transformation. DOSY was carried out using stimulated echo and LED by bipolar gradients for diffusion and two spoil gradients with water pre-saturation (ledbpgpr2s). Spectra were acquired using 16 scans per series of 8 K × 32 data points with linear gradients of 32 steps 2–96%, diffusion times of 0.1 s and the length of the square diffusion-encoding gradient pulses of 1 ms. The 2D-DOSY spectra were processed in the exponential mode on Topspin 3.2 (Bruker BioSpin Corporation, Billerica, MA, USA).

### EPS viscosity measurements by atomic force microscopy (AFM)

To measure viscosity, a silicon cantilever (160AC-NN, OPUS, NanoAndMore, Watsonville, CA, USA, *f*_res,air_ = 269 kHz, *k*_c_ = 28 N/m) with an Asylum MFP3D AFM (Oxford Instruments Asylum Research, Inc., Santa Barbara, CA, USA) was used and power density spectra (PDS) of thermally excited vibrations were recorded. At the resonance frequency *f*_res_, the spectra showed a maximum which either could be described by means of a single harmonic oscillator (SHO) (Van Eysden and Sader 2007) or by a Lorentzian peak shape (Franosch et al. 2011). To determine the viscosity of the extracts, a series of measurements on five mixtures of water and glycerine (2–20% v/v) were made to calibrate the sensing system.

### Supporting methods for AFM cantilever surface layer examination after fungal EPS contact

To demonstrate the corrosive nature of *K. petricola* A95 extracellular polysaccharides, a commercially available silicon cantilever (Pointprobe-Plus-NCHR, Nanosensors, Neuchatel, Switzerland: 125 µm length, 30 µm width, 4 µm thickness) covered with a thin film of aluminium (approximate film thickness < 10 nm), was immersed in solutions of the polysaccharides. An infrared laser beam (860 ± 20 nm) was directed onto the aluminium-covered reverse-side of the cantilever and reflected onto a photodiode. Comparative topography scans were performed with the Asylum MFP3D AFM in Tapping Mode. Aluminium dissolution from the cantilever surface was demonstrated with energy-dispersive X-ray analyses (EDX, EDAX Inc., NJ, USA); the electron energy was 15 kV (FEI XL 30, LaB6, Eindhoven, The Netherlands). The presence of adsorbed organic material on top of the aluminium layer was excluded by Confocal Raman Spectroscopy (custom-made by WITec, Ulm, Germany) of the immersed cantilever.

## Results and discussion

### Culture of *K. petricola* A95 and preparation of extracellular polysaccharides

Culture conditions at the border between solid surfaces and the atmosphere differ significantly from those in liquid culture. For example, when fungal cells are cultivated in liquid media, practically no EPS is directly observed and for this reason we decided to study the characteristics of EPS formed during sub-aerial growth. Although growth conditions favouring sub-aerial biofilm development yield only low amounts of EPS, this experimental setup should make the data relevant to the natural habitat of rock-inhabiting MCF.

**Fig. 1:**
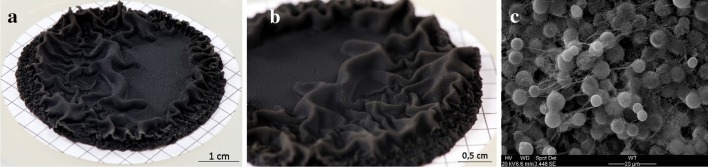
*K. petricola* A95 biofilms: **a** growing on cellulose acetate filters, **b** close-up view of the sub-aerial biofilm surface, **c** scanning electron microscopy of this biofilm surface

In contrast to the black yeast *A. pullulans* (which produces the EPS pullulan) that releases large amounts of EPS into its surroundings, the surface of A95 biofilms appears dry (Fig. 1). Most EPS are tightly bound to the cells and must be detached prior to analysis. Different chemical and physical extraction methods exist (Liu and Fang 2002; Pellicer-Nacher et al. 2013) but earlier work (Comte et al. 2006; Liu and Fang 2002) suggested that pre-treatment with formaldehyde and extraction with sodium hydroxide gave the highest extraction efficiency. Using these methods, the yields of crude *K. petricola* A95 EPS grown on malt-extract agar were about 103 mg g^−1^ cell dry weight.

### Fourier transformed infrared (FTIR) spectroscopy

**Fig. 2:**
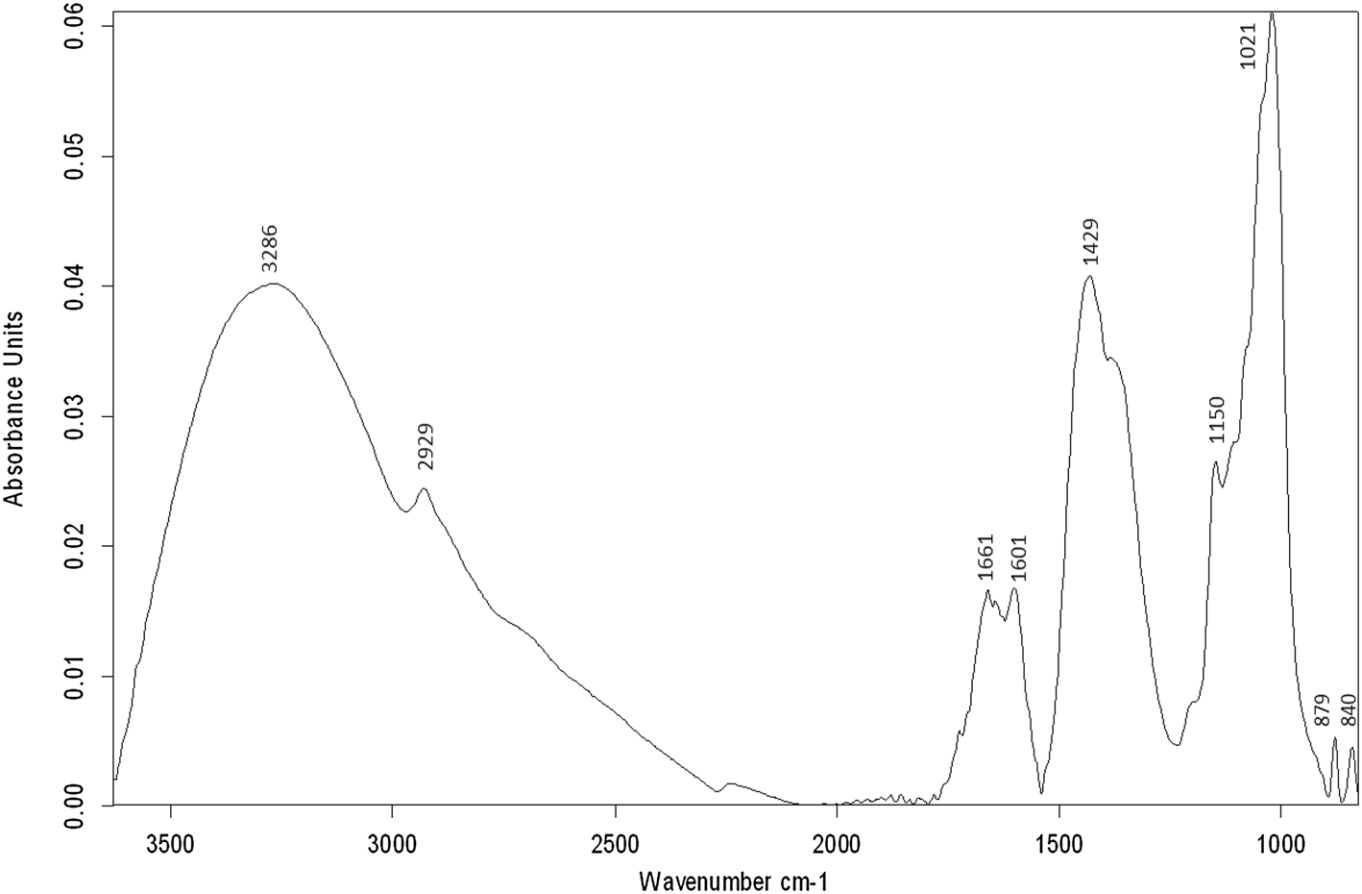
Fourier transformed infrared (FTIR) spectrum of EPS isolated from *K. petricola* A95. The spectrum represents the mean value of five recorded spectra, is baseline corrected and relieved of the CO_2_ peak

FTIR spectra of the crude EPS revealed typical functional groups (Fig. 2). A broadband around 3286 cm^−1^, represents the stretching vibration of both the hydroxyl groups of carbohydrates and amino groups of proteins (Fang et al. 2014; Liang et al. 2010). A weak absorption band at 2929 cm^−1^ is related to the asymmetrical C–H stretching vibration of aliphatic CH_2_-groups (Fang et al. 2014; Liang et al. 2010) while bands at 1658–1552 cm^−1^ were assigned to C=O, C=N and C–N of amide I and amide II vibrations in the presence of proteins (Fang et al. 2014). Other characteristic carbohydrate bands at 1440–1350 cm^−1^ represent the C–H bending of CH_2_ and CH_3_ (Mota et al. 2013). The C=O stretching vibration typical of uronic acids (1730 cm^−1^, if present), could not be detected (Mota et al. 2013). The strong band at 1021 cm^−1^ and a weak band at 1150 cm^−1^ were correlated with the C–O–C stretching vibration of carbohydrates (Fang et al. 2014). In addition to carbohydrates, these results revealed the presence of proteins and the absence of uronic acids in the EPS of *K. petricola* A95.

### Structure of *K. petricola* A95 extracellular polysaccharides

**Fig. 3:**
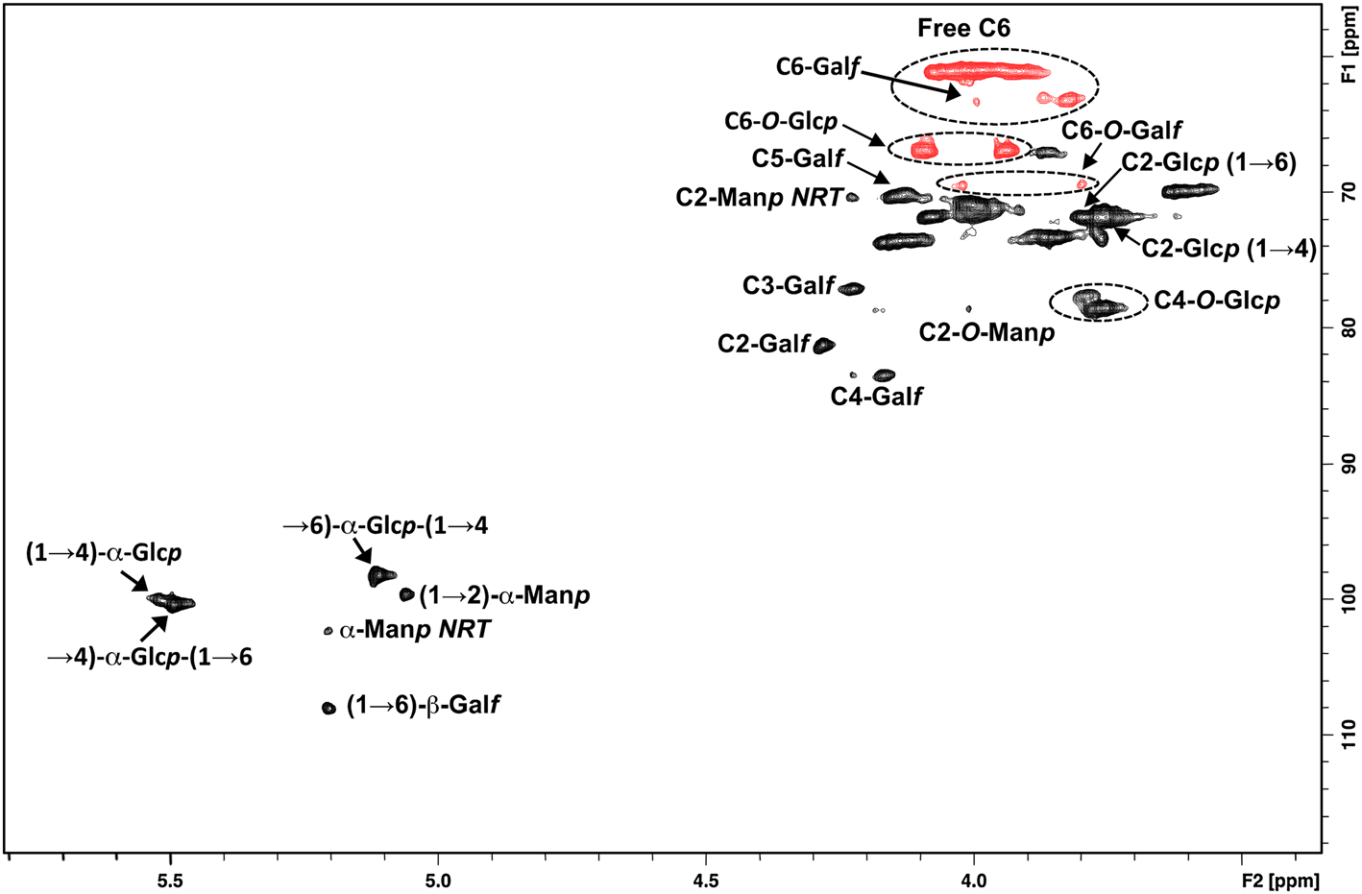
2-D-edited HSQC NMR spectra; the positive phase (black) corresponded to CH, and the negative phase (red) corresponded to CH_2_. ^1^H/^13^C NMR signals of *K. petricola* A95 extracellular polysaccharide mixtures determined by COSY and HSQC

**Fig. 4:**
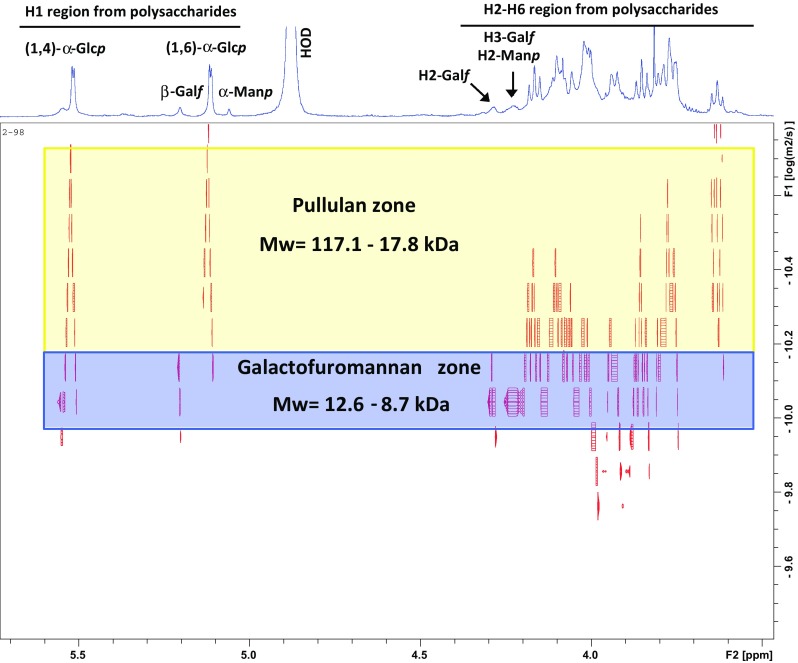
2-D-DOSY spectra of *K. petricola* A95 extracellular polysaccharides. ^1^H-NMR spectra—the chemical shifts are shown on the *x*-axis. Values on the *y*-axis represent the log of the diffusion coefficient obtained at 303 K. The main components are labelled in the spectra and the arrows cross the peaks in a specific log *D*

After hydrolysis, sugar residues were converted to alditol acetates. GC–MS analyses demonstrated that the extracted water-soluble polysaccharides mainly consisted of glucose (66.7%) with smaller amounts of mannose (20.6%) and galactose (12.7%), indicating a heteropolysaccharide. To characterise the glycosidic linkage pattern of the isolated polysaccharide, methylation analyses were performed. The presence of three different non-reducing end units—2,3,4,6-Me_4_-Glc*p* (7.8%), 2,3,5,6-Me_4_-Gal*f* (4.1%) and 2,3,4,6-Me_4_-Man*p* (1.6%) as well as the derivatives 2,4-Me_2_-Glc*p* (4.4%) and 2,6-Me_2_-Glc*p* (4.7%) were indicative of one complex branched heteropolysaccharide or more than one polymer (Table 1). High amounts of 2,3,6-Me_3_-Glc*p* (50.7%) and 2,3,4-Me_3_-Glc*p* (22.1%) suggest a main chain probably composed of (1,4)-linked glucopyranose and (1,6)-linked glucopyranose. Proportions of approximately 2:1 in the methylated alditol acetates of extracellular polysaccharides were also found in the extracellular α-glucan pullulan of the black yeast *A. pullulans* (Bender et al. 1959). Pullulan is a linear homo-polysaccharide comprising maltotriose trimers joined by α-(1,6) glycosidic linkages. The internal glucose units within maltotriose trimers are connected by α-(1,4) glycosidic bonds (Cheng et al. 2011). Only traces of (1,3)-linked glucopyranose, shown by the presence of the derivative 2,4,6-Me_3_-Glc*p*, could be found by methylation analyses. Other linkage types, including α-(1,3) and even β-(1,3) and β-(1,6) were found in the pullulan chain of different strains (Bouveng et al. 1962; Fujii et al. 1984; Sowa et al. 1963). The presence of 2,3,6-Me_3_-Glc*p* and 2,3,4-Me_3_-Glc*p* in similar proportions to those found in pullulan, indicates that *K. petricola* A95 mainly produces a pullulan-like polymer. It can also be concluded from the presence of 3,4,6-Me_3_-Man*p* (2.0%) and 2,3,5-Me_3_-Gal*f* (2.6%) (two strong signals at *δ* 5.493 and 5.109 shown in the ^1^H NMR spectra of extracellular polysaccharides giving ^3^ *J*_H1**–**H2_ of 3.84 and 3.68 Hz, respectively) that *K. petricola* A95 secretes a second polymer, composed of (1,2)-linked mannopyranose and (1,6)-linked galactofuranose. Small signals were also observed in the anomeric region at *δ* 5.058, 5.204 and 5.517 which suggest more than one polysaccharide was contained in the fraction. 2D-COSY analyses of vicinal couplings, mainly H1–H2 crossed peak correlations belonging to α-glucopyranosyl ring as far the other hydrogens of the molecules were determined (data not shown). Edited HSQC analyses were used to observe the glycosidic linkages and anomeric configuration of the polysaccharides in the mixture, of the main pullulan-like polysaccharide. The anomeric region of the polysaccharide showed characteristic signals with chemical shifts attributed to α-linked Glc*p* units at *δ* 100.3/5.493 and 100.0/5.516, corresponding to 4-O-substitution and at *δ* 98.2/5.109 that were attributed to 6-O-substituted units (Fig. 3). These O-substitutions influenced ^1^H and ^13^C chemical shifts and were confirmed due to the presence of signals at *δ* 78.5/3.768 and 77.6/3.790, which indicated direct substitutions at C-4 of the O-4. In the negative phase of HSQC, the C-6 substitution at *δ* 66.9/4.09, 3.937 was observed, corroborating the presence of α-(1,6)-linked glucopyranosyl units. The minor cross-peak correlation in the anomeric zone at *δ* 108.0/5.204 was attributed to β-Gal*f* units substituted at O-6, which was confirmed by detection of a negative phase signal at *δ* 69.4/4.021, 3.798. In addition, the correlations at *δ* 99.6/5.058 and 102.3/5.204 were typical of α-(1,2)-linked-Man*p*, belonging to the main chain and non-reducing terminal units, respectively (Fig. 3). Confirmation of C-2 substitution was obtained by the detection of the signal at *δ* 78.5/4.008 and the non-substituted C-2 of α-d-Man*p* non-reducing ends at *δ* 70.3/4.227, which agreed with previous studies (Kobayashi et al. 1995; Vinogradov et al. 1998). 2-D diffusion-ordered spectroscopy of (Fig. 4) showed a pullulan with different degrees of polymerisation (DP), since the correlations of 1H at *δ* 5.493 and 5.109 and log *D* had values ranging from − 10.56 to − 10.16, giving estimated molecular masses of 117.1–17.8 kDa, respectively. The heterogeneous profile of extracellular polysaccharides was also confirmed by HPSEC analyses (S1). Additionally, DOSY correlations at *δ* 5.204 and 5.058, which had log *D* values of − 10,089 to − 10,011, suggested an additional polysaccharide in the biofilm, a galactofuromannan with an estimated *M*_w_ varying ranging from 8.7 to 12.6 kDa. The latter probably consists of a branched galactofuromannan with a α-(1,2)-linked-Man*p* main chain attached by β-(1,6)-Gal*f* side chains, such as those found in the pathogenic yeast *Exophiala jeanselmei* (Sassaki et al. 2011). Thus, the extracellular polysaccharides of *K. petricola* A95 biofilms comprise two major components: a pullulan (80%) similar to that found in *Aureobasidium* (Dothideomycetes) and a galactofuromannan (20%) as in *Exophiala* (Chaetothyriales). It is interesting that *K. petricola* (Chaetothyriales) being a rock-inhabiting ancestor of *Exophiala* shares specific EPS traits with other members of the phylogenetically heterogeneous group of black yeasts (Gueidan et al. 2011; Ruibal et al. 2008).

**Table 1:** Partially *O*-methylated alditol acetates obtained by methylation analyses of *K. petricola* A95 extracellular polysaccharides

Partially *O*-methylated alditol acetates	*K. petricola* A95 (mol%)	Linkage type
2,3,4,6-Me_4_-Glc*p*	7.8	Glc*p*-(1 →
2,3,4,6-Me_4_-Man*p*	1.6	Man*p*-(1 →
2,3,5,6-Me_4_-Gal*f*	4.1	Gal*f*-(1 →
3,4,6-Me_3_-Man*p*	2.0	2 →)-Man*p*-(1 →
2,4,6-Me_3_-Glc*p*	Tr	3 →)-Glc*p*-(1 →
2,3,6-Me_3_-Glc*p*	50.7	4 →)-Glc*p*-(1 →
2,3,4-Me_3_-Glc*p*	22.1	6 →)-Glc*p*-(1 →
2,3,5-Me_3_-Gal*f*	2.6	6 →)-Gal*f*-(1 →
2,6-Me_2_-Glc*p*	4.7	3,4 →)-Glc*p*-(1 →
2,4-Me_2_-Glc*p*	4.4	3,6 →)-Glc*p*-(1 →

*Tr* traces

### Viscosity determination by atomic force microscopy

**Fig. 5:**
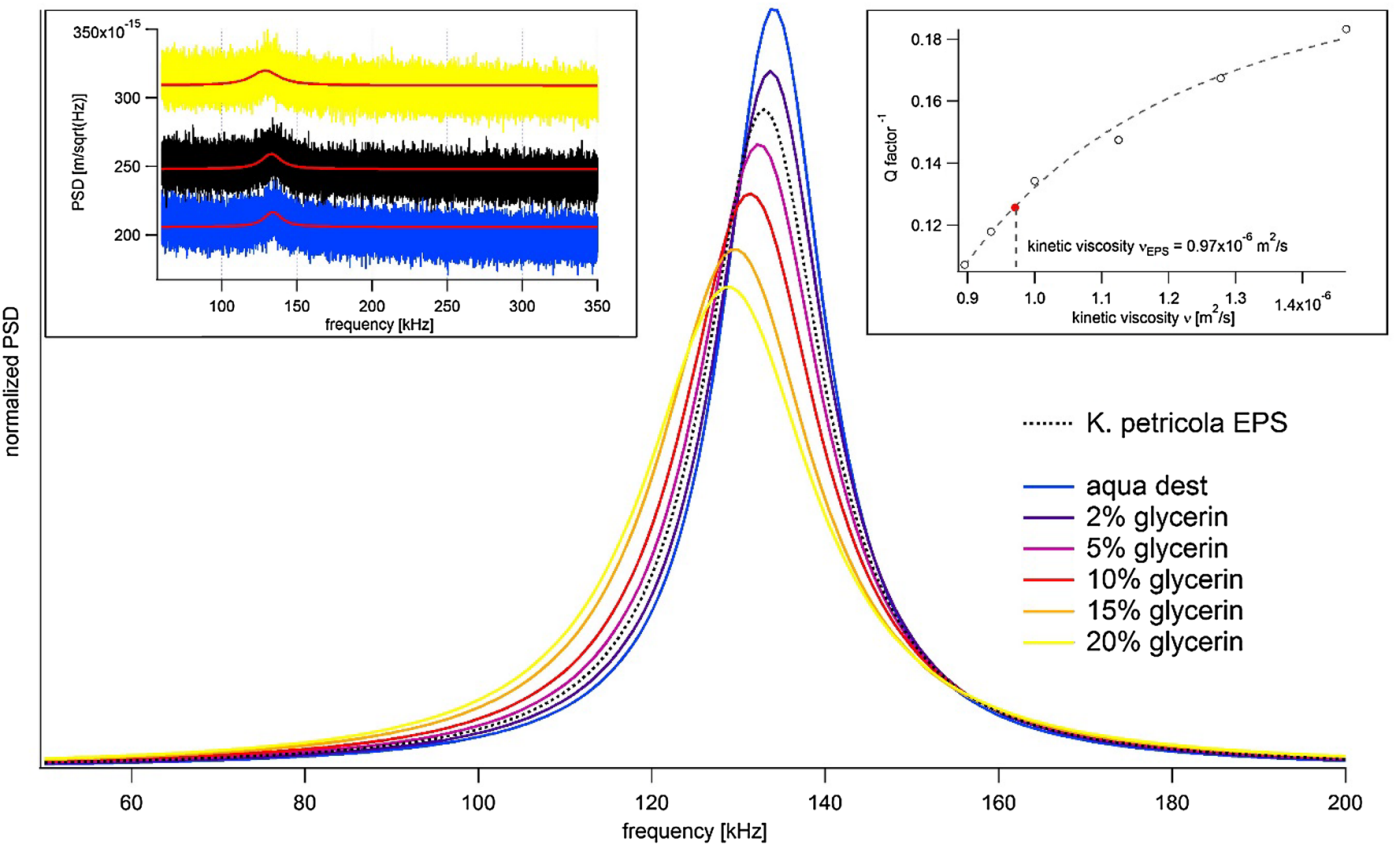
Lorentzian curves fitted to power spectra density plots (PSD) and normalised to peak area (i.e., corresponding energy). The left inset presents three examples for the fitting of the noise thermal spectra. Inverse *Q*-factor (i.e., damping) for six different glycerine/water mixtures and EPS with a fit describing *Q* ^−1^ (*ν*) is shown in the right inset. The kinetic viscosity of extracellular polysaccharides (red marker) was determined to be 0.97 × 10^−6^ m^2^ s^−1^

To estimate diffusion- and convection-controlled processes in EPS, it is first necessary to quantify the viscosity of EPS. Here, we used an AFM-based approach for two reasons. First, only small amounts of dissolved extracellular polysaccharides were available and AFM measurements require less than 200 µl of fluid. Second, micro-cantilever-based sensors act as nano-mechanical, thermally excited harmonic resonators and the monitored vibration resembles Brownian motion (Franosch et al. 2011), thus an undisturbed thermodynamic equilibrium can be assumed during measurements. Viscosity was quantified by recording power density spectra (PDS) of a thermally excited cantilever in liquid EPS. At a resonance frequency *f*_res_ of the cantilever, the spectra showed a maximum which can be described by means of a single harmonic oscillator (SHO) (Van Eysden and Sader 2007) or by a Lorentzian peak (Franosch et al. 2011). From a normalised Lorentzian fit, the quality factor *Q* of the cantilever could be calculated by *Q* = *f*_res_/2*Γ* with resonance frequency *f*_res_ and 2*Γ* as the full width at half maximum (FWHM) of the peak. The quality factor *Q* was inverse to the cantilever’s damping *D* and proportional to the kinematic viscosity $\nu = \frac{\eta }{\rho }$ (Tellechea et al. 2009), with *η* as the dynamic viscosity and the fluid density *ρ*. To calibrate the sensing system glycerine-in-water mixtures were chosen since viscosity and density for any given fraction of glycerine are known (Cheng 2008). With a kinetic viscosity ν of 0.97 × 10^−6^ m^2^ s^−1^, extracted *K. petricola* A95 extracellular polysaccharides ranged between 2 and 5% glycerine (Fig. 5).

### EPS-induced corrosion

**Fig. 6:**
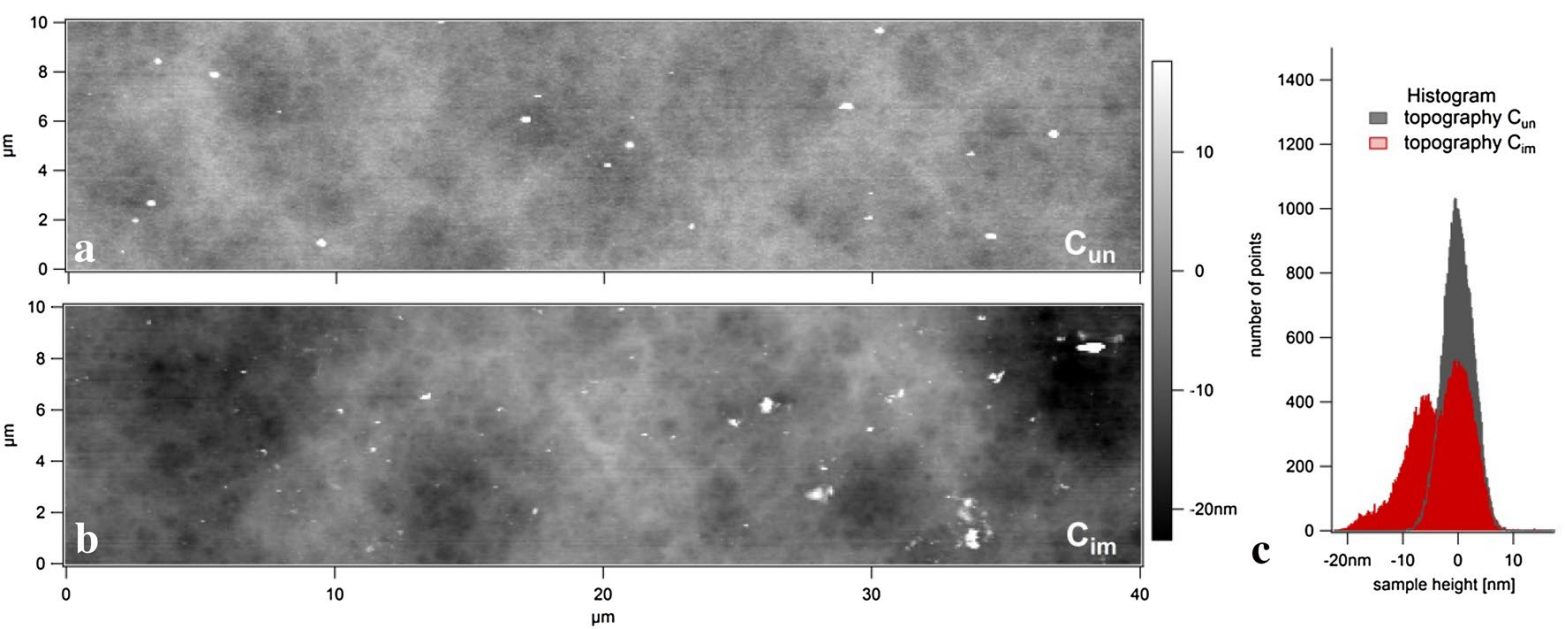
Topography of an unused (**a**, *C*_un_) and a used cantilever (**b**, *C*_im_) immersed for 1 h in *K. petricola* A95 extracellular polysaccharide solution. The histogram (**c**) demonstrates the increased surface roughness due to spatially selective corrosion (2nd left peak)

In AFM, the output signal of the photodiode is correlated to the intensity of the reflected laser beam—thus the output signal of the photodiode decreases if the reflectance of the cantilever declines. Aluminium coatings are commonly used to enhance the reflectance of the cantilever surface above that of silicon. The output signal in this setup was measured to be *U*_out_ > 7 V in air and < 3 V in aqueous media. The silicon cantilever covered with a thin film of aluminium significantly changed its properties after exposure to extracellular A95 polysaccharides. The cantilever was rinsed with water after 1 h contact with suspension, dried (< 1 h) and thereafter the reflectance of the surface dropped significantly (*U*_out_ < 4 V). Since absorbed material on the cantilever was not observed by Raman spectroscopy, displacement of the aluminium layer and changed reflection of the laser beam was expected. AFM topography scans of the previously immersed cantilever (compared to an unused cantilever) (Fig. 6a, b) verified the displacement revealed as well as drastic fluctuations in the aluminium layer thickness which could only be attributed to corrosion in the polysaccharide suspension (Fig. 6c).

**Fig. 7:**
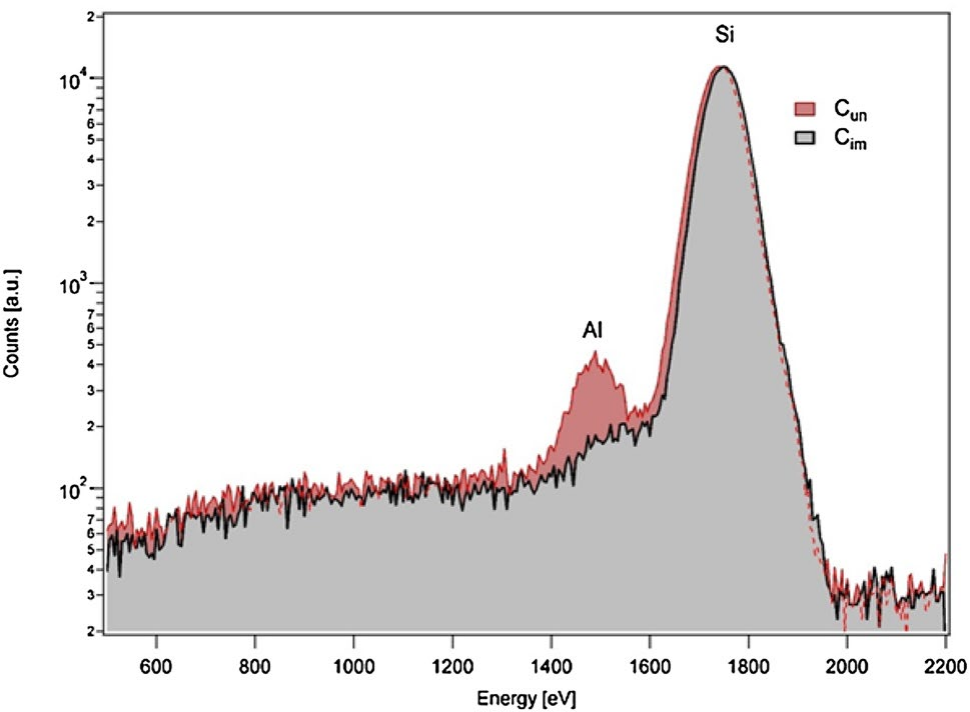
EDX spectra of *C*_un_ and *C*_im_ without background subtraction. The spectrum of *C*_im_ shows neither a signal for aluminium (AlK 1485 eV) nor for organic compounds (carbon, C–K 250 eV; oxygen, O–K 510 eV). Both curves were measured with identical sensitivities and integration times

Rather than a broadening of the peak at *t* = 0 nm, which would be typical for increased roughness, a second silicon surface at approx. *T* = − 7 nm was visible. At those cantilever points, aluminium was completely corroded and at some points the sample height was even below the apparent silicon surface (< *t* = − 10 nm). This would suggest that the silicon oxide layer was also corroded. EDX analyses showed that the Al layer was removed from the surface, leaving behind a silicon surface devoid of aluminium or its oxides (Fig. 7).

## Conclusions

We developed widely applicable methods for the extraction of EPS, their analysis and characterisation of extracellular polysaccharides from melanised rock-inhabiting stress-resistant fungi. Pullulan, an α-(1,4)-; α-(1,6)-glucan, was the main polysaccharide present (~ 80%) along with a branched galactofuromannan with a α-(1,2)-linked Man*p* main chain and a β-(1,6)-linked Gal*f* side chain (~ 20%). The kinetic viscosity of *K. petricola* A95 extracellular polysaccharides ranged between the equivalent of 2–5% (v/v) glycerine. EPS could thus have a profound effect on diffusion-dominated processes at material/SAB interfaces. Even within short exposures (≈ 1 h) the aluminium coatings of AFM cantilevers were significantly degraded by contact with extracellular polysaccharides of a rock-inhabiting fungal strain clearly demonstrating their corrosive powers. Interestingly, the corrosive properties were not dependent on uronic acids which were not found in the extracellular polysaccharides of *K. petricola* A95.

## Abbreviations

SAB: Sub-aerial biofilm
EPS: Extracellular polymeric substances
Gal*f*: Galactofuranose
Glc*p*: Glucopyranose
Man*p*: Mannopyranose
MCF: Microcolonial fungi
